# A pediatric case of *Chlamydia psittaci* caused severe Acute Respiratory Distress Syndrome (ARDS) in Italy

**DOI:** 10.1186/s13052-023-01497-6

**Published:** 2023-08-30

**Authors:** Serena Marchese, Giacomo Marchese, Giuseppe Paviglianiti, Maria Lapi, Gaetano Ottoveggio, Giuseppe Pipitone, Giovanni Corsello

**Affiliations:** 1 Emergency and Admission Medicine and Surgery, Childrens’Hospital, ISMEP-Arnas Civico-Benfratelli- Di Cristina, Palermo, Italy; 2 ASP Palermo (Provincial Health Authority), Palermo, Italy; 3 Pediatric Radiology, ISMEP-Arnas Civico-Benfratelli- Di Cristina, Palermo, Italy; 4 Anesthesia and Pediatric Resuscitation with Trauma Center, ISMEP-Arnas Civico-Benfratelli- Di Cristina, Palermo, Italy; 5 Systemic and immune-suppressed associated infection Disease Unit, INMI Lazzaro Spallanzani IRCCS, Rome, Italy; 6 Unit of General Pediatrics, Childrens’Hospital, ISMEP-Arnas Civico-Benfratelli- Di Cristina, University of Palermo, Palermo, Italy

**Keywords:** Psittacosis, Children, ARDS, SARS-COV-2, Dyspnea, Targeted antibiotic therapy, Medical history

## Abstract

**Background:**

This case of psittacosis in children, is the first described in literature, in Italy. This respiratory infection can be transmitted to humans from the inhalation of respiratory secretions, feces and plumage aerosol of infected birds (and other animals). Usually it can have an asymptomatic or paucisymptomatic course, and the onset is often flu-like, but in this case the child risked his life for a severe respiratory failure. This report is unique because in children psittacosis is rare, and always misdiagnosed, or could cause a delayed diagnosis because of lack of awareness among the paediatricians and physicians. Furthermore, psittacosis enters a differential diagnosis with *SARS-COV2* infection because both diseases may determine dyspnea and atypical pneumonia, up to acute respiratory failure.

**Case presentation:**

This clinical case talks about a three-and-a-half-year-old male child affected by psittacosis (or ornithosis), with severe dyspnea and systemic symptoms who required oro-tracheal intubation for acute respiratory failure. The child had slept in a room at home, with some recently bought parrots affected by psittacosis. Initially the child was treated with empiric antibiotic therapy (i.v.ceftriaxone and teicoplanin), but after having isolated the DNA of the germ “*Chlamydia psittaci*” in both serological and through bronchoalveolar lavage (BAL), he was treated with targeted antibiotic therapy: tetracyclines (doxicillin).

**Conclusions:**

Psittacosis is an extremely contagious disease, caused by an intracellular germ, called “*Chlamydia psittaci*”, a Gram-negative bacterium, transmitted to humans in particular by infected birds, responsible for atypical pneumonia, with acute and chronic respiratory symptoms, sometimes with multi-organ failure and disseminated intravascular coagulation. Even if it is a rare respiratory disease among children, a good doctor must think about psittacosis as cause of respiratory symptoms (and not only flu or *SARS-COV2*), above all through a correct medical history, in order to provide a targeted antibiotic therapy. An interesting case of psittacosis in a child is being reported here, which has been treated successfully with doxycillin.

**Supplementary Information:**

The online version contains supplementary material available at 10.1186/s13052-023-01497-6.

## Background

Psittacosis (or ornithosis) is an infection that can be transmitted to humans from the inhalation of respiratory secretions, feces and plumage aerosol of infected birds, hens, ducks and rarely pigs and horses [1].

The first cases of psittacosis transmitted by domestic parrots date back to 1879, when a Swiss physician, Jacob Ritter, described seven cases of atypical pneumonia, of which three were fatal, in his family and related them to the introduction into the family environment of some imported parrots and finches [2].

Although it is an ancient disease described in 1615 by Fra Bartolomeo [3], still there is no accurate diagnostic technique due to the intracellular nature of the pathogen.

Currently, there are multiple reports of new and unexpected cases of *chlamydia* associated with community-acquired pneumonia (CAP) around the world which highlight the importance of multi-disciplinary collaboration to tackle this pathogen. Around 1% of CAP worldwide is caused by *C. psittaci* [4].

*C. psittaci*, long considered to be the only pathogenic species in birds and aetiological agent of avian chlamydiosis and human psittacosis, is common in poultry farms worldwide. The disease severity in birds varies according to host species, age, and immune status as well as to the virulence of the bacterial strain [3, 5].

On psittacosis, in Italy since 2009, no epidemiological data has been received [25]. Up to date, this would be the first case of children psittacosis in Italy.

An Italian Study indicates a surprising percentage, over 8%, of antibodies anti-*Chlamydia Psittaci* in infants and children. This percentage varies little in relation to place of residence, rural or urban, or the presence of animals, but confirms the high risk in parrot-owning households where anti-Chlamydia antibodies are found in 37.5% of children [6].

In birds the infection is usually latent. In them, the disease can manifest itself in acute, subacute, and chronic forms with symptoms including anorexia, diarrhea, lethargy, weight loss, and sometimes it presents only mucopurulent or serious oculonasal discharge. In severe cases, dark green faeces, anorexia, dehydration, dyspnea, and death [7].

The worldwide prevalence of human ornithosis infection is low. Only a few cases of psittacosis have been described in the world, and only in adults.

Below we report the cases described in the literature. A systematic search of PubMed and Scopus databases of literature published between 01 January, 1986 and 03 July, 2017 was done and thirty-seven eligible articles were identified, describing 44 human psittacosis outbreaks in 12 countries. Laboratory tests performed were PCR (with various targets), serologic tests (complement binding reactions, ELISA’s, immunofluorescence tests and immuno-peroxidase tests) and culture, in various combinations. The literature provided no ‘gold standard’ laboratory testing strategy to identify recent human *C. psittaci* infections [8].

A study conducted in Germany based on real-time PCR analysis detected a higher percentage of patients positive for *C. psittaci* (2.1%) infection than *C. pneumoniae* (1.4%). Human infections from wild birds have not been widely documented [9].

In Belgium, the number of reported positive laboratory results increased slowly since 2010, and in 2017, the number almost doubled compared to the two previous years. Over the 3-year period, the mandatory notification system registered 24% only of all reported positive laboratory result [10].

In Argentina in 2017 María E.Cadario *et all.* described 8 human cases of psittacosis through respiratory samples and ocular swabs [11].

A recent case in China (2021) describes a 54-year-old woman with pneumonia and meningitis caused by *C. Psittaci*, who was treated by combined use of targeted antimicrobial agents of tetracyclines, macrolides and fluoroquinolones and was discharged home 28 days later, after admission to Intensive Care Unit. She required intubation and mechanical ventilation and the diagnosis was made by metagenome next-generation sequencing and clinical analysis [12].

In humans the infection penetrates the respiratory tract. Propagation takes place in the cells of the alveolar epithelium, epithelial cells of the bronchioles, bronchi, and trachea. Manifestations could be the destruction of the affected cells, the release of the pathogen, its toxins and cell decay products which, upon entering the blood, cause toxemia and sensitization [6, 13].

In severe cases, hematogenous drift of the pathogen in the parenchymal organs, central nervous system, myocardium etc., is possible [15]. In patients with reduced reactivity, the elimination of the pathogen is often delayed. It is in the cells of the endothelial reticulum, macrophages, epithelial cells of the respiratory tract for a long time. Under unfavorable conditions for microorganisms, the pathogen can enter the blood, causing systemic symptoms [14].

Psittacosis has an incubation period of 5–30 days (on average 7–14 days). It can have an asymptomatic or paucisymptomatic course, and the onset is often flu-like with fever, headache, cough, myalgia, asthenia, nausea, and vomiting [13]. Clinical symptoms are etherogeneous and could present both mild and severe cases: atypical pneumonia (mono or bilateral involvement), ARDS, hepatitis, glomerulonephritis, but also disseminated intravascular coagulation, septic shock, multi-organ failure, up to coma (European Commission 2002) [16, 17].

The laboratory tests are often non-specific and, especially in the paucisymptomatic forms, the white blood cells are often normal, with a slight increase in inflammation indices and of transaminases.

The diagnoses of psittacosis in humans is established based on clinical presentation and a positive serological result using microimmunofluorescence (MIF) with paired sera. MIF is generally more sensitive. More recently, molecular testing involving nucleic acid amplification, such as PCR, has increased in both reliability and availability [17]. These tests can be run on respiratory specimens, blood, and tissues, if warranted. In addition to being highly sensitive and specific for *C.psittaci*, nucleic acid–based tests can provide capacity for strain genotyping [17, 18].

The recommended antibiotic treatments for the psittacosis infection exist for adults. Tetracycline antibiotics are the first choice for treatment of human psittacosis. Mild to moderate psittacosis can be treated with doxycycline or minocycline orally, while severe disease needs to be treated with intravenous doxycycline. Generally, after treatment with tetracycline antibiotics, there is a response within 24–48 h, such as a decrease in body temperature. The course of medication should last at least 14 days, preferably up to 21 days, otherwise insufficient treatment will easily lead to relapse. Macrolide antibiotics, such as azithromycin, are regarded as the best alternative for patients with contraindications to tetracyclines. Fluoroquinolones, such as moxifloxacin and levofloxacin, have also been proven to be effective against *C. psittaci* [19].

To date, there are no recommendations for therapy of psittacosis in children, but they are rationally treated with tetracyclines for about 5–10 days orally, in particular doxicillin [20].

The main objective of this case report is to share in literature an uncommon case of pulmonary and systemic disease in children, which could be easily confused with every respiratory pathogenic germ, such as *SARS-COV-2*. In addition to this, it is essential to consider the environmental history, above all if the patient has respiratory distress of an unknown cause, in order to be treated in an appropriate manner and thus improve the prognosis [14].

## Case presentation

This chapter describes the case of a three-and-a-half-year-old male child, with persistent cough and severe dyspnea. These respiratory symptoms were present for 12 h (from the previous night). When the child got to the hospital, the main symptoms were: intercostal, subcostal and jugular re-entries (refractory to drug therapy), severe desaturation (88%), perioral cyanosis and no fever. On auscultation of the chest, whistles and hisses were appreciated on the right lung (bronchospasm-like) and hypophonesis on the left one. He performed a molecular swab for *SARS-COV-2* which gave negative results. He was given oxygen at 10 L per minute (FiO2 50%) by face mask, and 15 mg of iv methyl prednisone. Its weight was 15 kilos. Epinephrine was administered by aerosol and also salbutamol with beclomethasone-dipropionate, with no benefit.

**Fig. 1:**
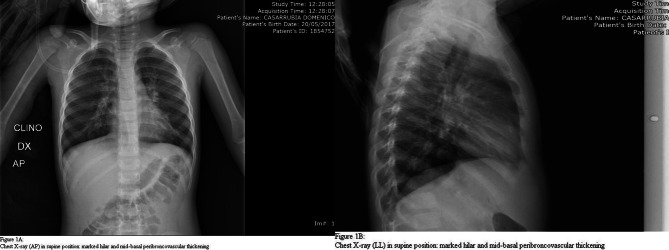
Chest X-ray (AP) in supine position. (**A**) Marked hilar and mid-basal peribroncovascular thickening. (**B**) Marked hilar and mid-basal peribroncovascular thickening

On the chest X-ray (2p) taken in supine position, given the clinic, it is highlighted: “enlargement of the iloperilar regions with peribronchovasal thickening at the mid-basal fields with prevalence left, in hyper-expanded lungs. Heart shadow within limits " (Fig. 1A, B).

There were no radiographic signs compatible with the inhalation of a foreign body, nor cardiomegaly to suggest a myocarditis.

After above-described various therapeutic approaches (beta2-agonist, adrenaline, salbutamol and beclomethasone dipropionate for aerosol, iv methyl prednisone and O2 therapy) the resuscitator was alerted with whom the urgent hospitalization in Intensive Care Unit for respiratory failure refractory to medical therapy was agreed.

Blood chemistry tests at entry: arterial blood gas analysis pH 7.13, paO_2_ 85, paCO_2_ 78, BE -3.3, HCO_3_- 26, P / F 85, LAC 1.1. Neutrophilic leukocytosis (WBC 16 × 10^3^ cells / uL, N 13.6 × 10^3^ cells / uL), CRP (C-reactive protein) 3.66 mg / dL, PCT 0.97 ug / L, LDH 286 IU / L, Fibrinogen 443 mg / dL (Table 1).

**Table 1:** Laboratory tests

Laboratory tests	molecular swab for *SARS-COV-2*	DNA of *Chlamydia Psittaci* (BAL and serological tests)	WBC cells/uL	N cells/uL	CRP mg/dL	PCT ug/L	LDH IU/L	Blood gas arterial analysis
**Admission**	negative	positive	16 × 10^3^	13.6 × 10^3^	3.66	0.97	286	pH 7.13, paO_2_ 85, paCO_2_ 78, BE -3.3, HCO_3_- 26
**Discharge**	negative	/	8 × 10^3^	2,5 × 10^3^	0,3	0	80	pH 7,44, paO_2_ 78 mmHg, paCO_2_ 50, HCO_3_ 32,3, BE 9,8

This table shows improvement of laboratory tests from hospital admission to discharge, after targeted antibiotic therapy (tetracyclines)

Absence of impaired coagulation and liver and kidney function markers [34].

After one hour of hospitalization in Pediatric Intensive Care unit (PICU), given the clinical worsening, the little patient underwent an Oro-tracheal Intubation (IOT) and a Mechanical Assisted Ventilation (VAM), after sedation and curarization. In the following hours there was a worsening of pulmonary exchanges resulting in a severe ARDS picture that required protective ventilation.

Therefore, broad spectrum empirical antibiotic therapy (iv ceftriaxone and teicoplanin), sedation and continuous curarization were started [15].

**Fig. 2:**
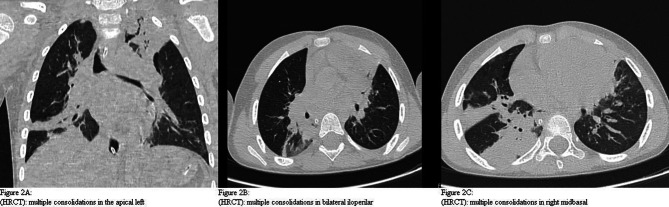
Chest HRCT. (**A**) Multiple consolidations in the apical left. (**B**) Multiple consolidations in bilateral iloperilar. (**C**) Multiple consolidations in right midbasal

Following the critical evolution of the respiratory picture, in the following 6 hours, he had a lung CT examination (HRCT) with 16-layer MDCT (Siemens) which highlighted a clearly worsening evolution of the radiological picture with a mixed pattern (interstitium-alveolar) : “scattered consolidations, the major ones of which in the left upper apical area, in the middle fields in the iloperilar, and right posterior basal (with aerial bronchograms); an interstitial-reticular pattern associated with small areas of ground glass; small amount of right posterobasal pleural effusion and borne by the small fissure " (Fig. 2A, B, C).

On the second day of hospitalization, the little sister (1.5 years old) was also hospitalized in the Infectious Diseases Department of the same hospital, for mild dyspnea and neutrophilic leukocytosis; to follow, the brother (10 years) and the cousin (1.5 years) also for the same symptomatology.

At this point, deepening the anamnesis in search of environmental risk factors, it was clear that the parents had bought some parrots about a week before the first child was admitted and that everyone had played and even slept in the same room with them.

Thus, the research in the first child admitted to PICU, both serological (immunofluorescence tests) and through bronchoalveolar lavage (BAL), of the DNA of *Chlamydia Psittaci* (PCR analysis) started, with a positive result which therefore confirmed the picture of psittacosis.

They were all finally treated with targeted antibiotic therapy: tetracyclines (doxicillin) [18, 21].

The siblings and cousin are discharged after a few days, thanks to timely targeted antibiotic therapy.

As regards to our first child admitted to PICU, after targeted antibiotic therapy (doxicillin), gas exchanges improved, with a net reduction in dyspnea and oxygen requirements, so extubation was carried out on the fifth day.

During the days of hospitalization, blood gas analyzes were performed every day, with progressive improvement in respiratory exchanges. The child went through respiratory acidosis (pH 7,13, paO_2_ 85 mmHg, paCO_2_ 78 mmHg, HCO_3_ 25,9, BE-3,3) to a normal respiratory condition on the sixth day (pH 7,44, paO_2_ 78 mmHg, paCO_2_ 50, HCO_3_ 32,3, BE 9,8).

Therefore, a detailed anamnestic investigation, together with the clinical-radiological evolutionary picture, directed the diagnosis towards psittacosis pneumonia with evolution into ARDS.

The etiological picture was confirmed with laboratory tests (isolation of the bacterium from clinical samples, search for antibodies) [18].

## Discussion and conclusions

In the dyspnoeic child, in the suspicion of pneumonia or to exclude foreign body inhalation, the emergency pediatrician may find it useful to request a thoracic X-ray which remains the first reference examination to make differential diagnosis in the respiratory patient. Currently, all patients who access the emergency area with respiratory symptoms must be considered potentially *SARS-COV-2* +, and undergo screening with nasopharyngeal swabs for the molecular search for the aforementioned virus.

Generally, in a pediatric setting, chest X-ray is performed in the AP (supine) or PA (standing) view only. Lateral projection can be performed in selected cases at the explicit request of the radiologist. In hospitalized patients, chest x-ray examination in bed is a valid tool for the evolutionary monitoring of pneumonia [22].

Sometimes, as in the reported case, it is necessary to perform a chest CT examination, which must be reserved for clinically selected patients: clinical-radiological discrepancy, patients who are not responsive to drug therapy or who show clinical deterioration. CT also discriminates the pulmonary alterations that are sometimes underestimated radiographically with greater accuracy.

Chest ultrasound (POCUS - Point of Care UltraSound), performed at the patient’s bed, is an aid in therapeutic diagnostic monitoring and can reduce excessive use of diagnostic imaging [19].

As in other forms of atypical pneumonia (*SARS-COV-2, Mycoplasma Pneumoniae, Chlamydia Pneumoniae,  Legionella*), even in psittacosis, there are no pathognomonic radiological patterns but some, from a review of the literature, are characteristic:

- areas of consolidation in the basal and central sites appear to be frequent [22–24] 
- a pattern of thickening of the peribronchial interstitium often coexists with striae and lattices 
- 80% of patients have a migratory evolution of infiltrates also in other lobes [14] 
- a small percentage have a ground-glass framework (HRCT), or non-specific anomalies due to increased density [21] 
- according to some authors, the ground glass “Halo Sign”, which surrounds some nodular-shaped consolidation areas, although not specific, characterizes some parenchymal alterations in psittacosis [24–26, 30] 
- atelectasis or pleural effusions rarely occur and, if present, they are small 
- the extent of the consolidations, when present, correlate with the severity of the clinical picture [25] 

Chlamydial infections continue to be underestimated and underreported in both poultry and human sectors worldwide [25]. To date, the infection is not routinely investigated as part of the diagnosis panel in case of respiratory diseases and pneumonia in humans [27]. In Italy, avian chlamydiosis due to *C. psittaci* infection is included in the animal notifiable diseases list, and psittacosis is included among notifiable occupational diseases.

On psittacosis, in Italy since 2009, no epidemiological data has been received [28]. The only cases reported in Italy are co-infections in chronic diseases (like polyarthritis and psoriasis) [28, 29].

Up to date, this is the first Italian case of psittacosis in children. The only case of a child affected by psittacosis described in literature is in Pakistan (2016), and he was treated successfully with azithromycin [14].

During the *COVID-19* pandemic, to the pediatric emergency department, we noticed an increase in the numbers of acute respiratory failure (children aged from 1 month to 3 years), probably due to the lack of immunological memory (main cause: constant use of face mask). Our experience with all these cases, brought us to establish that not only *COVID-19* can be the infectious agent, but there are many other germs, above all respiratory viruses, and more rarely *Chlamydia Psittaci*, which have almost the same clinical presentation (dyspnea, cough and atypical pneumonia). The treatments are different and must be initiated as soon as possible, to avoid bad prognosis.

The main causes of ARDS in pediatric age (pARDS) are represented by viral infections (*RSV, Adenovirus,  Influenza, and Parainfluenza viruses*, etc.) [14, 31].

The exceptionality of this case is given precisely by the very rare etiology in the pediatric field and by having changed a poor prognosis into a favorable outcome, thanks to the in-depth medical history, early diagnosis and a targeted antimicrobial therapy.

This represents the first Italian case of psittacosis described in children.

In conclusion, it is very important, above all during the *SARS-COV-2* pandemic, not to dwell on the suspected viral pneumonia (like *SARS-COV-2*, *RSV, Adenovirus, Influenza, and Parainfluenza viruses*, etc.) but to investigate also the environmental anamnesis to prevent other serious respiratory diseases with even worse prognoses [31, 32, 34, 35].

Although acute psittacosis with severe acute respiratory failure is unusual, especially in children, knowing the clinical, anamnestic and radiological picture of this disease, among the possible diagnoses of atypical pneumonia, can save the lives of our little patients [33, 35].

### Electronic supplementary material

Below is the link to the electronic supplementary material.

Supplementary Material 1

Supplementary Material 2

## Data Availability

The datasets used and/or analyzed during the current study are available from the corresponding author on reasonable request.

## List of Abbreviations

MIF: Microimmunofluorescence
WBC: White blood cells
N: Neutrophil ratio
CRP: C-reactive protein
PCT: Procalcitonin
i.v.: Intravenous
PICU: Pediatric Intensive Care unit
IOT: Oro-tracheal Intubation
VAM: Mechanical Assisted Ventilation
BAL: Bronchoalveolar lavage
POCUS: Point of Care UltraSound
ARDS: Acute respiratory distress syndrome
pARDS: Pediatric acute respiratory distress syndrome
RSV: Respiratory syncytial virus
HRCT: High-resolution computed tomography
CT: Computed axial tomography
AP: Antero-posterior
PA: Postero-anterior
MDCT: Multidetector computed tomography
LDH: Lactate dehydrogenase
BE: Base excess
paO_2_: Partial pressure oxygen
paCO_2_: Partial pressure carbon dioxide
HCO_3_: Bicarbonate
