# Supplementary material for: A pediatric case of Chlamydia psittaci caused severe Acute Respiratory Distress Syndrome (ARDS) in Italy
Source: Ital J Pediatr. 2023 Aug 30;49:107. doi: 10.1186/s13052-023-01497-6 (PMC10468848; doi:10.1186/s13052-023-01497-6)
Supplement: Supplementary file 1 — Supplementary Material 1 [file 13052_2023_1497_MOESM1_ESM.docx]

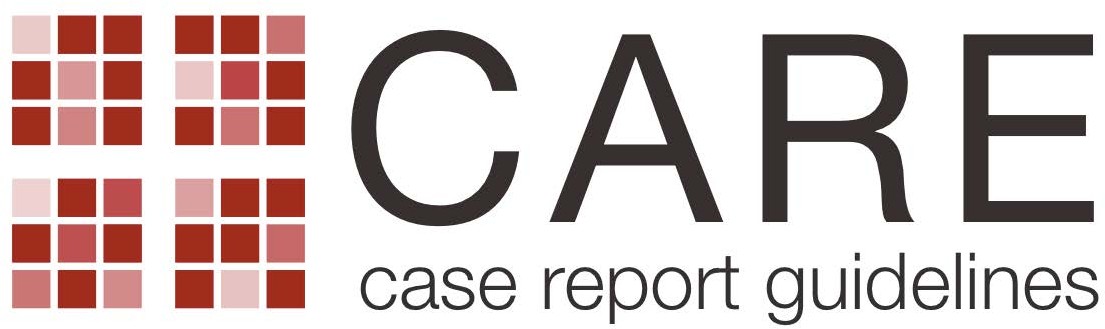
CARE Checklist of information to include when writing a case report
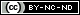


**Topic Item Checklist item description Reported on Line**

**Title 1 1** A pediatric case of severe Acute Respiratory Distress Syndrome (ARDS) resembling SARS-COV-2. It was psittacosis!

Case report

**Key Words 2** Psittacosis, children, ARDS, SARS-COV-2, dyspnea, targeted antibiotic therapy, medical history, case report

# Abstract

**(no references)**

**3a**  This case report is the first described in literature, among children in Italy. It is unique because in children psittacosis is

rare, and always misdiagnosed, instead in this child caused ARDS.

**3b** Severe dyspnea and systemic symptoms who required oro-tracheal intubation for acute respiratory failure

**3c** At the beginning, severe dyspnea required oro-tracheal intubation. After having isolated the DNA of the germ

“Chlamydia psittaci” in both serological and through bronchoalveolar lavage (BAL), he was treated with tetracyclines

(doxycillin) with rapid improvement of the general clinical conditions.

**3d** Take home messages: Even if it is a rare respiratory disease among children, a good doctor must think about psittacosis

as cause of respiratory symptoms (and not only flu or SARS-COV2), above all through a correct medical history, in order

to provide a targeted antibiotic therapy

**Introduction 4** On psittacosis, in Italy since 2009, no epidemiological data has been received. Up to date, this would be the first

case of children psittacosis in Italy. An Italian Study indicates a surprising percentage, over 8%, of antibodies anti-

Chlamydia Psittaci in infants and children. This percentage varies little in relation to place of residence, rural or urban, or

the presence of animals, but confirms the high risk in parrot-owning households where anti-Chlamydia antibodies are

found in 37.5% of children.

*Musso A, Riva C, Balbo L, Valpreda A, Garbaccio P, Migliore G, Zannino Anti-Chlamydia psittaci antibodies in a*

*healthy pediatric population. L.Minerva Pediatr. 1991 Apr;43(4):305-9.PMID: 1870537 Italian.*

**Patient Information 5a** three-and-a-half-year-old male child, with persistent cough and severe dyspnea for 12 hours. The child had slept in a

room at home, with some recently bought parrots affected by psittacosis

**5b** persistent cough and severe dyspnea for 12 hours, not responsive to any therapy

**5c** Healthy child that slept at night in a room at home, with some recently bought parrots affected by psittacosis

**5d** Relevant past interventions with outcomes: none

# Clinical Findings

**Timeline**

**Diagnostic Assessment**

**Therapeutic Intervention**

**Follow-up and Outcomes**

1. When the child arrived at the hospital, the main symptoms were: intercostal, subcostal and jugular re-entries (refractory to drug therapy), severe desaturation (88%), perioral cyanosis and no fever. On auscultation of the chest, whistles and hisses were appreciated on the right lung (bronchospasm-like) and hypophonesis on the left one. He performed a molecular swab for SARS-COV-2 which gave negative results.
2. Historical and current information from this episode of care organized as a timeline

**8a** molecular swab for SARS-COV-2 which gave negative results. chest X-ray (2p) taken in supine position. Blood

chemistry tests at entry: arterial blood gas analysis. Resuscitation couseling.

**8b** Diagnostic challenges (such as access to testing, financial, or cultural): none

**8c** Diagnosis of psittacosis, after excluding covid disease.

**8d** none

**9a** He was given oxygen at 10 liters per minute (FiO2 50%) by face mask, and 15 mg of iv methyl prednisone. Its weight

Was 15 kilos. Epinephrine was administered by aerosol and also salbutamol with beclomethasone-dipropionate, with no

benefit.  Oro-tracheal Intubation (IOT) and Mechanical Assisted Ventilation (VAM), after sedation and curarization.

Empirical antibiotic therapy (iv ceftriaxone). They were all finally treated with targeted antibiotic

therapy:tetracycline (doxycillin).

**9b** Oro-tracheal Intubation (IOT) and Mechanical Assisted Ventilation (VAM) for 5 dys, doxycillin

**9c** At the beginning he was treated with Empirical antibiotic therapy (iv ceftriaxone). After having isolated the DNA of the

Germ “Chlamydia psittaci” in both serological and through bronchoalveolar lavage (BAL), he was treated with

Tetracyclines (doxycillin)

**10a** Clinician and patient-assessed outcomes : The child went through respiratory acidosis (pH 7,13, paO2 85 mmHg, paCO2 78

mmHg, HCO3 25,9, BE-3,3) to a normal respiratory condition on the sixth day (pH 7,44, paO2 78 mmHg, paCO2 50,

HCO3 32,3, BE 9,8).

**10b** None

**10c** None

**10d** Adverse and unanticipated events: none

**Discussion 11a** the strengths: to have rapidly isolated Chlamydia Psittaci and to have began targeted antibiotic therapy (doxycillin). AND

limitations: it was necessary to ask immediately for the environmental anamnesis given the severe dyspnea not responsive to any therapy

**11b** There is little literature on psittacosis and it is dated as well

**11c** The exceptionality of this case is given precisely by the very rare etiology in the pediatric field and by having changed a

poor prognosis into a favorable outcome, thanks to the in-depth medical history, early diagnosis and a targeted

antimicrobial therapy.

This represents the first Italian case of psittacosis described in children.

**11d** In conclusion, it’s very important, during the SARS-COV-2 pandemic, not to dwell on the suspected Coronavirus

pneumonia, but to thoroughly investigate also the environmental anamnesis to prevent other serious respiratory diseases

with even worse prognoses than SARS-COV-2 (20).

Although acute psittacosis with severe acute respiratory failure is unusual, especially in children, knowing the clinical, anamnestic and radiological picture of this disease, among the possible diagnoses of atypical pneumonia, can save the lives of our little patients.

*Maffei C, Marracino A, Di Stanislao F, Pauri P, Clementi M, Varaldo PE. Psittacosis in a highly endemic area in Italy. Epidemiol Infect. 1987 Oct;99(2):413-9. doi: 10.1017/s095026880006790x. PMID: 3315709; PMCID: PMC2249287*

**Patient Perspective 12** yes

**Informed Consent 13** Did the patient give informed consent? Please provide if requested . . . . . . . . . . . . . . . . . . . . . . . . . . . . . . . . . . . . . . **Yes x No**
