# Supplementary material for: A pediatric case of Chlamydia psittaci caused severe Acute Respiratory Distress Syndrome (ARDS) in Italy
Source: Ital J Pediatr. 2023 Aug 30;49:107. doi: 10.1186/s13052-023-01497-6 (PMC10468848; doi:10.1186/s13052-023-01497-6)
Supplement: Supplementary file 2 — Supplementary Material 2 [file 13052_2023_1497_MOESM2_ESM.odt]

COVER LETTER
I was forced to rejec the previous manuscript only for health reasons (I got infected with Covid-19) and I had no time to change the mistakes ok the manuscript. And I promised my colleagues that I would take care of the changes.
MS ID of the original submission was ITJP-D-21-00267.
This case presentation talks about a three-and-a-half-year-old male child affected by psittacosis (or ornithosis), with severe dyspnea and systemic symptoms who required
oro-tracheal intubation for acute respiratory failure. We know that this disease is rare in children, and the uniqueness of this case report is exactly the severity and rapid worsening of symptoms that initially thought to be attributable to sars-cov-2 (during the epidemic covid-19). The exceptionality of this case is given precisely by the very rare etiology in the pediatric field and by having changed a poor prognosis into a favorable outcome, thanks to the in-depth medical
history, early diagnosis and a targeted antimicrobial therapy.
This represents the first Italian case of psittacosis described in children. 


POINT BY POINT RESPONSE TO REVIEWERS:


1) I modified the English, also thanks to the help of an English Professor (Jonathan Mills ) whom we thanked a lot in the manuscript.
The symptoms were similar to the severe form of covid-19, ad we at the Pediatric Emergency room in Palermo have had some cases.

ABSTRACT

1) I moved some information in the introduction.
2) I removed the references in the abstract
3) I transformed “consequence” in manifestations

INTRO
Pag 2
1) I specified that we were talking of psittacosis
2) I inserted in the first paragraph general and epidemiology information (unfortunately there is little literature on psittacosis in children) and way of transmissions
3) Some of the general information were moved to the intro

4) I modifed the English in line 45-51
5) 56-57 I modifed the English
6) We were wong to write in the plural, having described a single case, even if we quickly made a reference to the other three relatives of the child concerned who presented themselves to the emergeney room, but who had milder symptoms
7) I modifed the English of the final paragraph of the intro

CASE REPORT
Pag 3
1)  I modifed the English of the whole case report
2) I inserted the duration of symptoms befor at Emergency room and the anamnesis information. He had not fever (I wrote it).
3) Line 33 I inserted what kind and how muh oxygen was given
Pag 4
4) line 7 I inserted what kind of antibiotic was started
5) I eliminated the table of the evolution of blood gas exchange
DISCUSSION

Pag 4 I modified the discussion and, following the last reviewer, I have combined the discussion with the conclusions.

CONCLUSION

Reviewer 2 Thank you so much to have appreciated our manuscript and above all the importance of a good and depth medical history.
I modified the unclear sentences.
